# The Goodyear Vulcanite Co. and the Rubber Patents

**Published:** 1881-02

**Authors:** R. Finley Hunt


### THE GOODYEAR DENTAL VULCANITE CO., AND THE RUBBER PATENTS.

The following letter, which explains itself, we take from *Items of Interest* for January.

WASHINGTON, D. C., Jan. 3, 1881.

T. B. WELCH, M. D. Dear Sir: Your esteemed favor of 29 ult. to hand. I am glad that you wrote to me, because I believe that I am better acquainted with all the points of the rubber patents, their history and the litigations about them, than any other dentist in the country. This knowledge has been gained from a persistent fight against the Cummings' patent (which I have always believed to be a fraudulent one) for *sixteen years* past. This sixteen years fight has crippled me seriously in my finances and practice, but I have as an offset two facts to console me: 1st, That my resistance to the unjust claims of the Goodyear Dental Vulcanite Company has caused them to expend not less than $100,000. 2d, And more important, that in this sixteen years fight I have collected and am in possession of sufficient material in the shape of evidence, etc., to enable me, if properly backed and assisted by the profession, to prevent the G. D. V. Co., getting an extension of the Cummings' patent by special act of Congress or saddling us with the Haering patent.

*They can do either of these if there is no organized and proper opposition.* With reference to the former I have been watching the proceedings of Congress last session and this in order to apprise the Profession as soon as any move is made in that direction, but it *should be organized and prepared before the battle comes on.* The move by Dr. Sibley is in the right direction, but does not go far enough in this, that the petitions should be backed up by *evidence,* that the patentee has been amply remunerated for his alleged invention, that the present owners have also been amply remunerated, and that the patent is not now in the hands of the alleged inventor, and such other points as may have a bearing on the case.

With reference to the Haering patent, your informant seems to have been somewhat astray. The following are the facts: Robert Haering filed in the Patent Office an application for a patent March 25, 1856, which was rejected. Oct. 14, 1868, he assigned his invention to John B. Newbrough, of New York, and obtained a patent (No. 85,927) Jan. 19, 1860, and John B. Newbrough, in a compromise agreement with the G. D. V. Co., agreed to convey and assign to said G. D. V. Co., this invention and patent of Haering, if they should be decided by the courts to be legal and valid. This compromise agreement bears date July 1st, 1869. The following extract from an address made by me to a meeting of dentists in Philadelphia, in 1872, will show what I thought of it then: "It (the Haering patent) is now believed to be owned by the Goodyear Dental Vulcanite Co., who, if successful now, will, in all probability, tie *it* to the Cummings' patent as they tied *that* to the Goodyear, and thus perpetuate tax upon dentists for fourteen wearisome years from this date."

You will thus see that your informant was not at all posted as to facts, and that the G. D. V. Co., is better fortified than you supposed. I think that an organized, united and *well directed* effort will be required to defeat the Co. I am willing to do all I can in any way, but I can do very little unaided. I would like to see the Profession of the country organized in each State by *Congressional Districts* for the purpose of influencing Representa tives and raising funds for expenses, and the conduct of the defence of our rights placed in the hands of a well selected general committee. An inefficient defence has hitherto only added strength to the G. D. V. Co. Yours truly,

R. Finley Hunt.

